# Intrapapillary Hemorrhage With Adjacent Peripapillary Subretinal Hemorrhage and Reduced Blood Flow in the Optic Papillary Region Observed on Laser Speckle Flowgraphy

**DOI:** 10.1155/crop/2726088

**Published:** 2026-02-24

**Authors:** Mizuki Ikeda, Toshiyuki Oshitari, Tomohiko Usui

**Affiliations:** ^1^ Department of Ophthalmology, International University of Health and Welfare Narita Hospital, Narita, Japan; ^2^ Department of Ophthalmology and Visual Science, Chiba University Graduate School of Medicine, Chiba, Japan, chiba-u.ac.jp

**Keywords:** incomplete posterior vitreous detachment, intrapapillary hemorrhage with adjacent peripapillary subretinal hemorrhage, laser speckle flowgraphy, myopia, tilted optic disc

## Abstract

Intrapapillary hemorrhage with adjacent peripapillary subretinal hemorrhage (IHAPSH), commonly observed in myopic eyes with tilted optic discs, typically resolves without treatment. Although IHAPSH cases have been rarely reported, no prior studies have assessed laser speckle flowgraphy (LSFG) in this context. We report a case of IHAPSH evaluated using LSFG. A 54‐year‐old woman presented with floater symptoms in her right eye upon awakening. She had no history of trauma or medication use. Her best corrected visual acuity (BCVA) was 1.2 in both eyes. Fundus examination and optical coherence tomography revealed a tilted disc with adjacent peripapillary subretinal hemorrhage surrounding the disc hemorrhage. After 1 month, the hemorrhages partially resolved without treatment, and complete resolution was observed after 3 months, with BCVA remaining at 1.2 in the affected eye. The mean blur rate obtained from LSFG showed mild improvement following a temporary decline in the affected eye but remained lower than that in the unaffected left eye. This case underscores the potential utility of LSFG in evaluating IHAPSH and suggests that reduced local blood flow around the disc following the initial disruption may prevent recurrence, even under conditions of elevated blood pressure.

## 1. Introduction

Intrapapillary hemorrhage with adjacent peripapillary subretinal hemorrhage (IHAPSH) is a rare condition [1, 2]. It involves acute bleeding within the optic disc and adjacent peripapillary subretinal regions. IHAPSH is predominantly observed in myopic eyes with tilted optic discs and spontaneously resolves, with recurrence being exceedingly uncommon. This condition predominantly affects Asian individuals. Kokame et al. reported that patients with IHAPSH are often women, and the susceptible ages range from teenagers to older adults, whose average is 47 years [1]. The mechanisms underlying IHAPSH remain controversial, and the factors contributing to the absence of recurrences after the initial event are not well understood. In this case report, we describe a patient with IHAPSH evaluated using laser speckle flowgraphy (LSFG; Softcare Co. Ltd., Fukutsu, Japan). LSFG is a noninvasive technique that measures and visualizes blood flow in multiple blood vessels simultaneously, providing insights beyond those obtained with conventional imaging devices [3]. To our knowledge, this is the first report of IHAPSH examined using LSFG. We also discuss potential reasons for the absence of recurrence following the initial disruption of the vessel walls surrounding the optic disc.

## 2. Case Report

**Figure 2:**
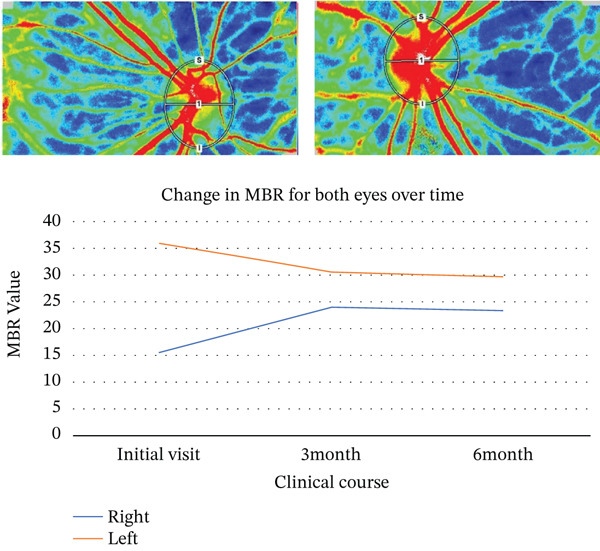
The mean blur rate (MBR) assessed by laser speckle flowgraphy (LSFG) in both eyes. The rubber band was positioned as shown in the photograph, and the measurements were consistently performed within the same region throughout all sessions. Mild improvement in the MBR was observed in the right eye. The MBR in the right eye was smaller than that in the left eye throughout the follow‐up period.

**Figure 4:**
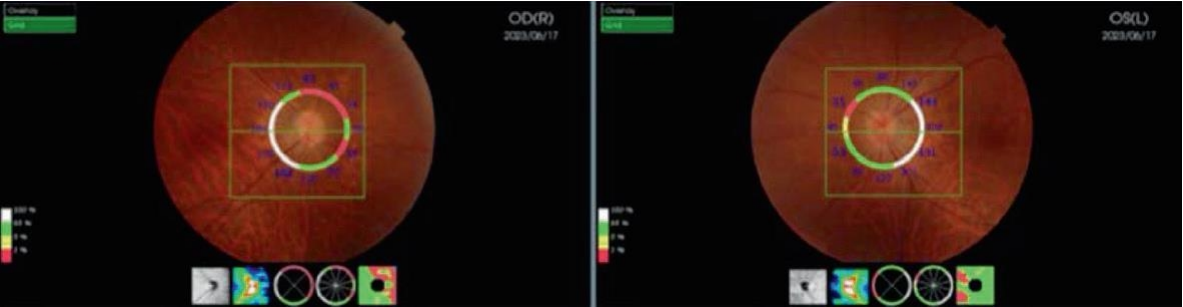
Circumpapillary optic disc analysis from OCT findings in both eyes. Hypoplasia of the nasal sides of both optic discs was presented, but the nasal retinal nerve fiber layer thickness in the right optic disc seems to be slightly thinner than that in the left optic disc.

A 54‐year‐old woman presented with floater symptoms in her right eye upon awakening. She had no history of trauma or medication use. Her best corrected visual acuity (BCVA) was 1.0 in the right eye (OD) and 1.2 in the left eye (OS), with intraocular pressures of 18 mmHg in the right eye and 17 mmHg in the left eye. Refractive examinations revealed −7.0 diopters OD and −5.5 diopters OS. Slit‐lamp examinations showed no abnormalities in the anterior segments of either eye. Critical fusion frequencies were 34 Hz in the right eye and 37 Hz in the left eye. Fundus and optical coherence tomography (OCT) examinations identified a tilted disc, hemorrhages in the optic disc and peripapillary subretinal space, and mild vitreous hemorrhage in the right eye (Figure 1a). No findings of peripapillary hyperreflective ovoid mass‐like structure (PHOMS) were observed around the optic disc from OCT examination (Figure 1b). Fluorescein angiography revealed blocked fluorescence due to peripapillary subretinal hemorrhage during the early phase, without definite leakage or new vessel formation in the late phase (Figure 1c,d). The Goldmann perimeter showed an enlarged Mariotte blind spot (Figure 1e (e‐1 and e‐2)). LSFG demonstrated a reduced mean blur rate (MBR) in the right eye. Blood tests and brain magnetic resonance imaging (MRI) revealed no abnormalities. After 1 month, the hemorrhages partially resolved without treatment (Figure 1f (f‐1 and f‐2)), and complete resolution was observed after 3 months (Figure 1g (g‐1 and g‐2)), with BCVA improving to 1.2 OD. The MBR showed mild improvement following an initial decline in the affected eye but remained lower than that in the left, unaffected eye (Figure 2). The tilt angle of the optic disc measured by OCT was 12.005° in the right eye and 11.086° in the left eye (tilt angle ratio: 1.08) (Figure 3). Circumpapillary optic disc analysis from OCT findings showed that the nasal side of the right optic disc hypoplasia was slightly worse than the left optic disc, but the asymmetry was not so large between both eyes (Figure 4).

Figure 1Clinical findings of the case. (a) A color photograph and (b) an optical coherence tomography (OCT) image of the right eye at the first visit showing hemorrhages in the optic disc and the peripapillary subretinal space. (c, d) Fluorescein angiography revealed blocked fluorescence due to peripapillary subretinal hemorrhage during the early phase and no leakage in the late phase. (e) e‐1 and e‐2: The Goldmann perimeter demonstrated an enlarged Mariotte blind spot in the left eye. (f) f‐1 and f‐2: After 1 month, the hemorrhages had partially resolved, and (g) g‐1 and g‐2: after 3 months, no recurrence of hemorrhages was observed.

**Figure:**
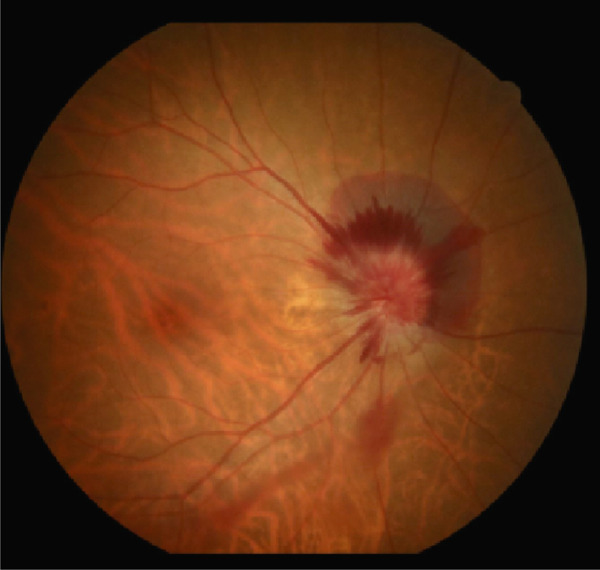
(a)

**Figure:**
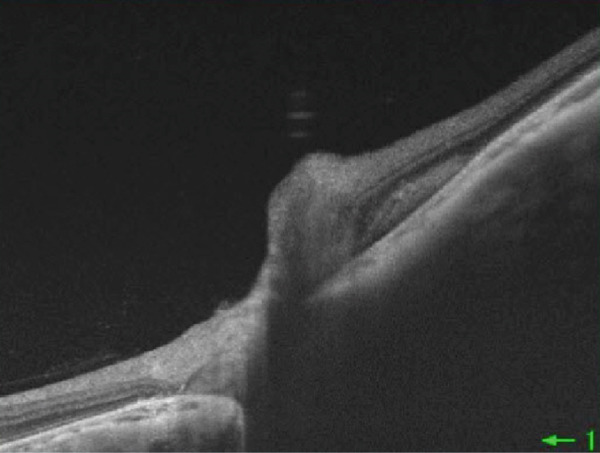
(b)

**Figure:**
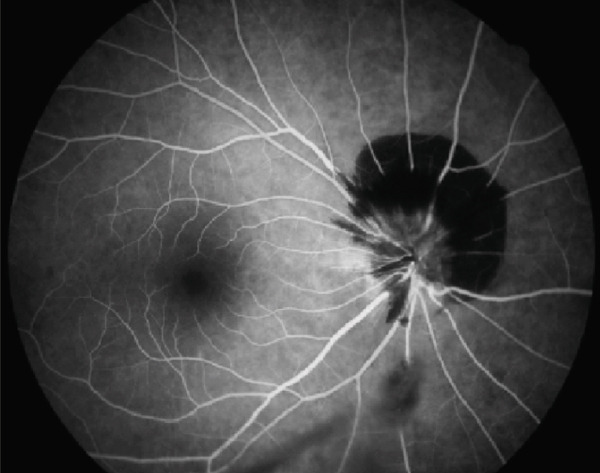
(c)

**Figure:**
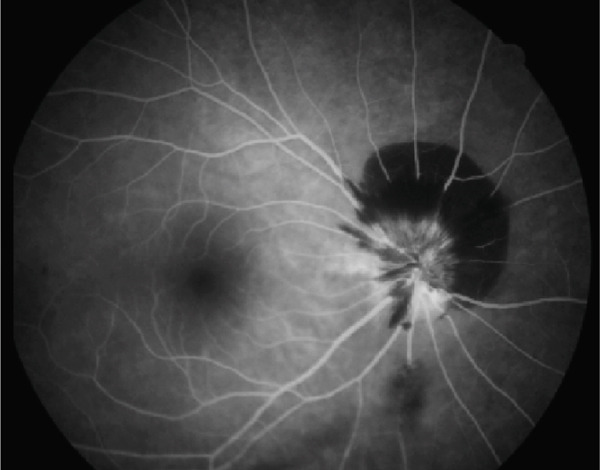
(d)

**Figure:**
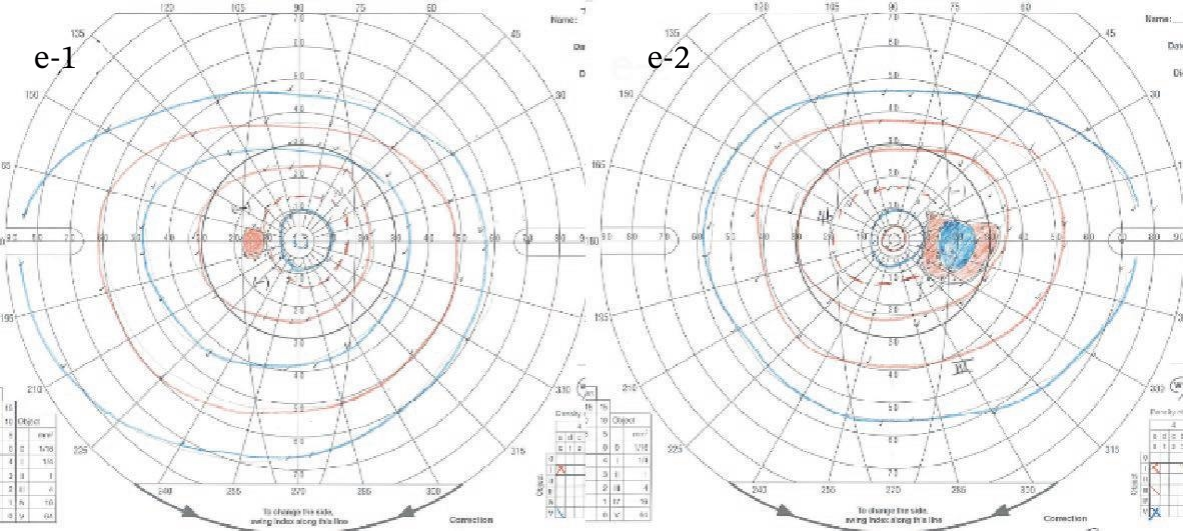
(e)

**Figure:**
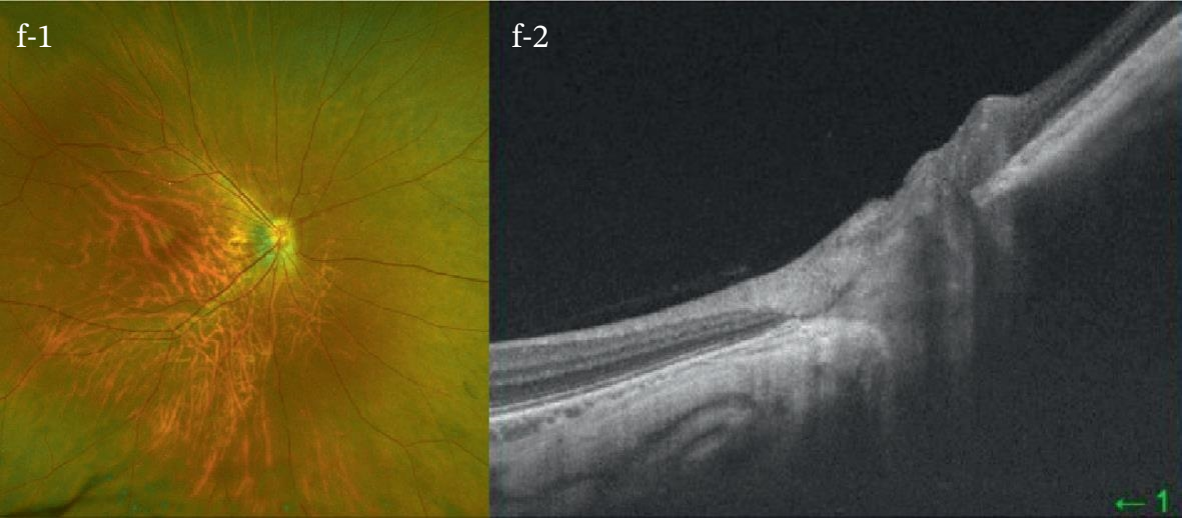
(f)

**Figure:**
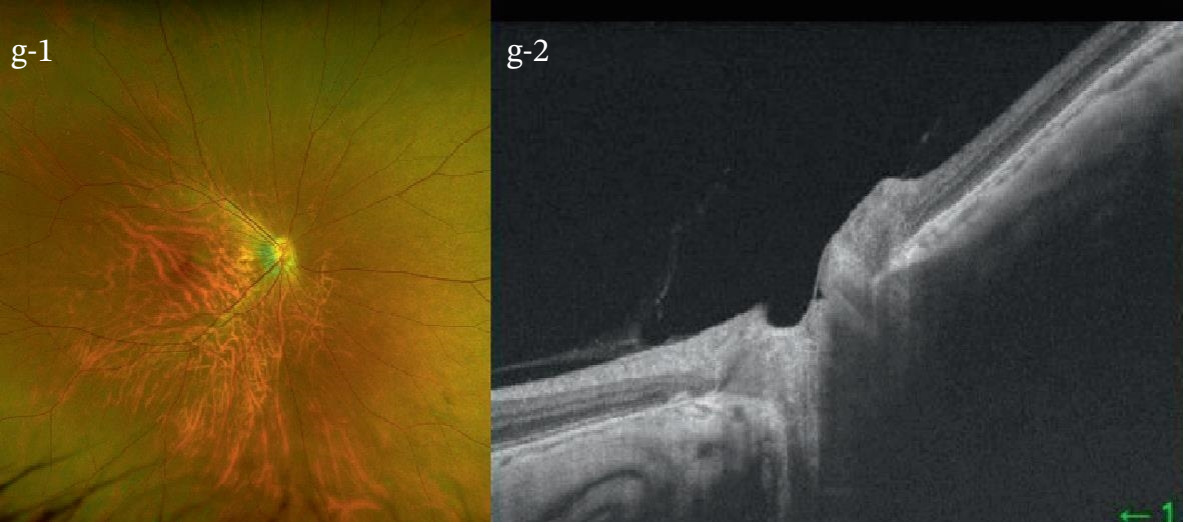
(g)

Figure 3OCT B‐scan images. The tilt angle is (a) a‐1 and a‐2: 12.005° in the right eye and (b) b‐1 and b‐2: 11.086° in the left eye (the tilt‐angle ratio: 1.08).

**Figure:**
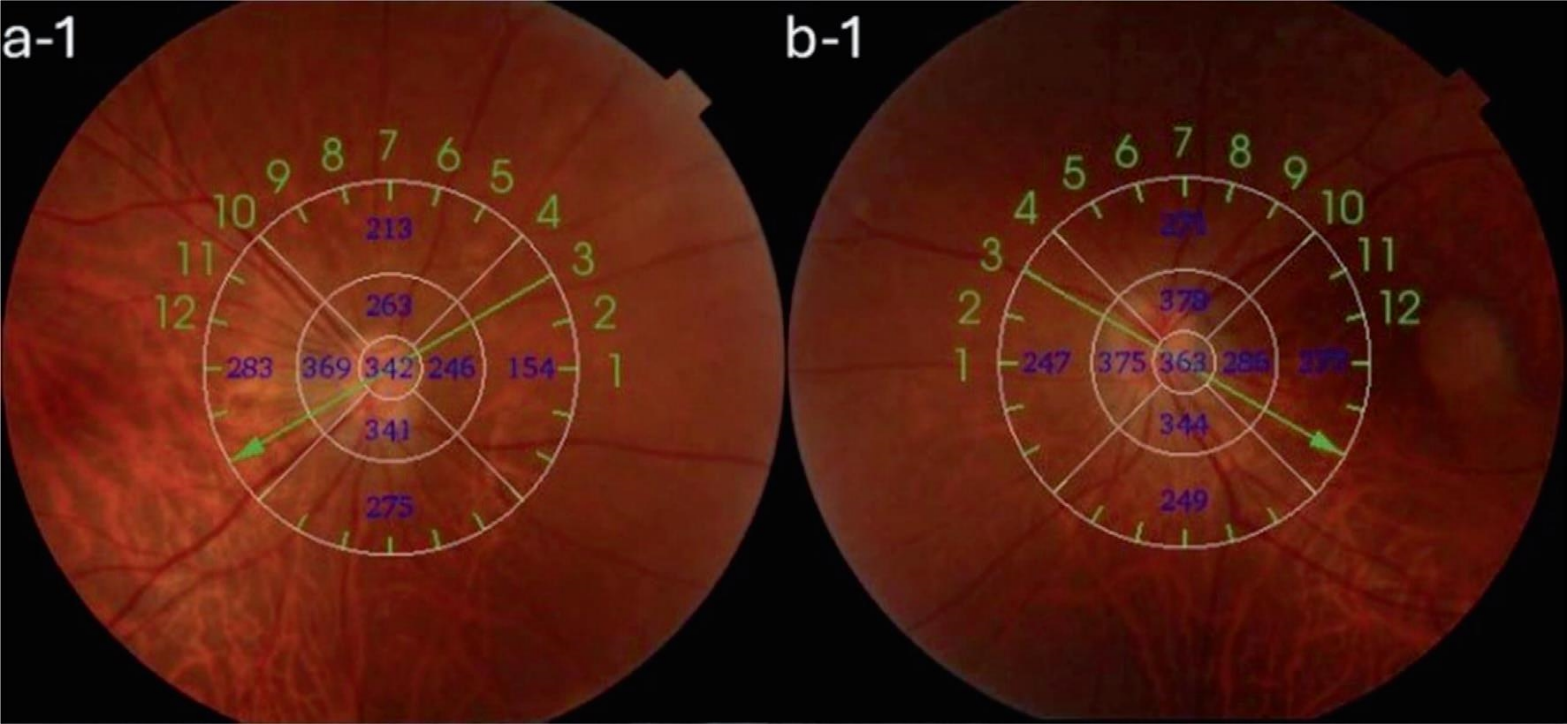
(a)

**Figure:**
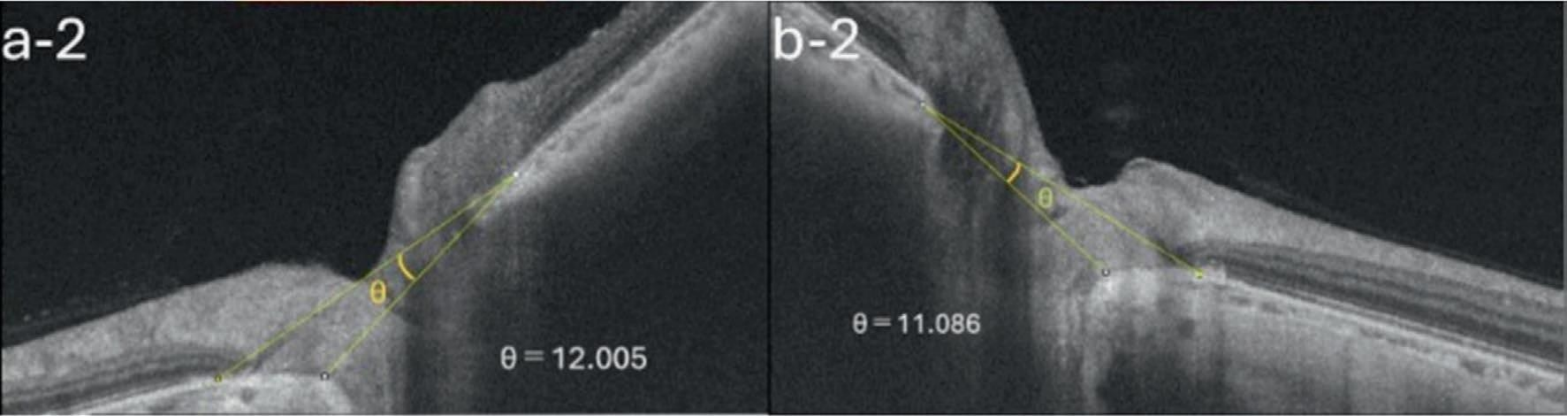
(b)

## 3. Discussion

IHAPSH commonly occurs in eyes with a tilted disc associated with myopia and generally follows a benign clinical course with a good visual prognosis. Hemorrhages in the optic disc and peripapillary subretinal space typically resolve spontaneously with no apparent damage to the optic nerve or retina. Accurate diagnosis is crucial to avoid unnecessary treatment, as IHAPSH must be differentiated from conditions including optic neuritis, polypoidal choroidal vasculopathy, optic disc drusen, disc hemorrhage, and other chorioretinal vascular diseases.

Key features of IHAPSH include (1) bleeding from the optic disc; (2) higher prevalence in eyes with tilted discs, particularly in myopia; (3) frequent involvement of the nasal and superior aspects of the optic disc; (4) acute onset with spontaneous recovery of good visual prognosis; (5) no recurrence; and (6) predominant occurrence in Asian women [1, 4–8]. However, the mechanisms underlying IHAPSH remain controversial, though several hypotheses have been proposed in the literature. One compelling hypothesis suggests horizontal stretching of the choroid and Bruch′s membrane at the choroidal papillary border, resulting in anatomical weakness due to ocular axis elongation and tilted papilla, which may stress the vasculature. Other hypotheses include hemorrhage from anatomically vulnerable prelaminar blood vessels in tilted optic discs, vitreopapillary traction, complications from edematous changes of the optic disc, and hemodynamic effects of the Valsalva maneuver [2, 4]. The prelaminar region receives arterial blood supply from the posterior short ciliary arteries and peripapillary choroidal arteries, whereas venous drainage occurs predominantly through the central retinal vein and is partly supplied from peripapillary choroidal veins [5, 9, 10]. Circadian periodicity in blood pressure may act as a stimulus for IHAPSH development [8]. Furthermore, optic disc traction from incomplete vitreous detachment is a well‐supported theory explaining vessel disruption surrounding the optic disc [4]. Previous studies have reported that a greater angle of optic disc tilt is associated with the development of IHAPSH [11]. In the present case, the eye affected by IHAPSH also showed a larger tilt angle of the optic disc compared to the unaffected eye.

In this case, LSFG provided significant insights. LSFG is a fundus blood flow imaging device using the laser speckle phenomenon to assess blood flow in the fundus, with the velocity of the blood flow evaluated as the MBR [12, 13]. The patient demonstrated mild improvement in the MBR after a temporary decline in the affected eye with IHAPSH. However, the MBR was reduced compared with that in the contralateral unaffected eye. Horizontal compression and stretching of the choroidal papillary area due to the tilting of the papilla, combined with optic nerve papilla traction caused by incomplete posterior vitreous detachment and blood pressure fluctuations such as early morning elevation, may have resulted in vitreous and subretinal hemorrhages. Subclinical blood flow reduction, as demonstrated by LSFG, was observed after the initial hemorrhages. This localized blood flow reduction may decrease the risk of vessel wall disruption surrounding the optic disc, potentially explaining the absence of recurrence despite increased blood pressure.

## 4. Conclusion

IHAPSH commonly occurs in eyes with myopic eyes, accompanied by a tilted disc architecture, and typically follows a benign clinical course with a good visual prognosis. Based on LSFG results, the absence of IHAPSH recurrence may be attributed to a mild reduction in local blood flow surrounding the optic disc, which potentially lowers the risk of vessel wall disruption during blood pressure fluctuations.

## Author Contributions

M.I. saw the patients, gathered data, and wrote the draft. T.O. and T.U. helped to write the draft and edited the final manuscript.

## Funding

This study was funded by a grant‐in‐aid from the Ministry of Education, Science, Sports, and Culture of the Japanese Government (22K09829) and a grant‐in‐aid from the Eye Research Foundation for the Aged in Japan.

## Disclosure

All of the authors had no commercial relationships.

## Consent

The patient provided consent for the use of her clinical details and scans for publication purposes.

## Conflicts of Interest

The authors declare no conflicts of interest.

## Data Availability

The data that supported the findings of this study are available from the corresponding author upon reasonable request.

## References

[1] Kokame G. T. , Yamamoto I. , Kishi S. , Tamura A. , and Drouilhet J. H. , Intrapapillary Hemorrhage With Adjacent Peripapillary Subretinal Hemorrhage, Ophthalmology. (2004) 111, no. 5, 926–930, 15121370, 10.1016/j.ophtha.2003.08.040, 2-s2.0-2342585526. 15121370

[2] Kokame G. T. , Intrapapillary, Peripapillary, and Vitreous Hemorrhage, Ophthalmology. (1995) 102, no. 7, 1003–1004, 9121741, 10.1016/s0161-6420(95)30923-2, 2-s2.0-0029329712. 9121741

[3] Tomita R. , Iwase T. , Fukami M. , Goto K. , Ra E. , and Terasaki H. , Elevated Retinal Artery Vascular Resistance Determined by Novel Visualized Technique of Laser Speckle Flowgraphy in Branch Retinal Vein Occlusion, Scientific Reports. (2021) 11, 20034, 10.1038/s41598-021-99572-7. 34625616 PMC8501139

[4] Katz B. and Hoyt W. F. , Intrapapillary and Peripapillary Hemorrhage in Young Patients With Incomplete Posterior Vitreous Detachment: Signs of Vitreopapillary Traction, Ophthalmology. (1995) 102, no. 2, 349–354, 10.1016/S0161-6420(95)31018-4, 2-s2.0-0028893975. 7862424

[5] Moon I. H. , Lee S. C. , and Kim M. , Intrapapillary Hemorrhage With Concurrent Peripapillary and Vitreous Hemorrhage in Two Healthy Young Patients, BMC Ophthalmology. (2018) 18, no. 1, 10.1186/s12886-018-0833-z, 2-s2.0-85049921699. PMC6045832 30005697

[6] Zhang X. , Cheng X. , Chen B. , and Sun X. , Multimodal Imaging Characteristics and Presumed Cause of Intrapapillary Hemorrhage With Adjacent Peripapillary Subretinal Hemorrhage, Clinical Ophthalmology. (2021) 15, 2583–2590, 10.2147/OPTH.S304861. 34177259 PMC8219308

[7] Teng Y. , Yu X. , Teng Y. , Xu B. , Sun Q. , Dong L. , Su Y. , Wu X. , and Dai B. , Evaluation of Crowded Optic Nerve Head and Small Scleral Canal in Intrapapillary Hemorrhage With Adjacent Peripapillary Subretinal Hemorrhage, Graefe′s Archive for Clinical and Experimental Ophthalmology. (2014) 252, no. 2, 241–248, 10.1007/s00417-013-2459-4, 2-s2.0-84894222259, 24057175. 24057175

[8] Yan C. L. , Brelén M. E. , Chen H. , and Chen W. , Spontaneous intrapapillary hemorrhage with adjacent peripapillary subretinal hemorrhage in adolescents, Clinical and Experimental Vision and Eye Research. (2019) 2, no. 1, 6–10, 10.15713/ins.clever.21.

[9] Hayreh S. S. , The Blood Supply of the Optic Nerve Head and the Evaluation of It- Myth and Reality, Progress in Retinal and Eye Research. (2001) 20, no. 5, 563–593, 10.1016/S1350-9462(01)00004-0, 2-s2.0-0034928732. 11470451

[10] Onda E. , Cioffi G. A. , Bacon D. R. , and Van Buskirk E. M. , Microvasculature of the Human Optic Nerve, American Journal of Ophthalmology. (1995) 120, no. 1, 92–102, 10.1016/S0002-9394(14)73763-8, 2-s2.0-0029036132. 7611333

[11] Takahashi S. , Kawashima R. , Morimoto T. , Sakimoto S. , Shiozaki D. , Nishida K. , Kawasaki R. , Sakaguchi H. , and Nishida K. , Analysis of Optic Disc Tilt Angle in Intrapapillary Hemorrhage Adjacent to Peripapillary Subretinal Hemorrhage Using Swept-Source Optical Coherence Tomography, American Journal of Ophthalmology Case Reports. (2022) 27, no. 27, 101598, 10.1016/j.ajoc.2022.101598. 35651596 PMC9149012

[12] Kobayashi T. , Shiba T. , Okamoto K. , Usui T. , and Hori Y. , Characteristics of Laterality in the Optic Nerve Head Microcirculation Obtained by Laser Speckle Flowgraphy in Healthy Subjects, Graefe′s Archive for Clinical and Experimental Ophthalmology. (2022) 260, no. 9, 2799–2805, 10.1007/s00417-022-05631-8, 35298697. 35298697

[13] Takeshima S. , Higashide T. , Kimura M. , Udagawa S. , Yamada Y. , Takemoto D. , Ohkubo S. , and Sugiyama K. , Effects of Trabeculectomy on Waveform Changes of Laser Speckle Flowgraphy in Open Angle Glaucoma, Investigative Ophthalmology & Visual Science. (2019) 60, no. 2, 677–684. 30786279 10.1167/iovs.18-25694

